# DLK1 Expressed in Mouse Orexin Neurons Modulates Anxio-Depressive Behavior but Not Energy Balance

**DOI:** 10.3390/brainsci10120975

**Published:** 2020-12-12

**Authors:** Tatiyana Harris, Raluca Bugescu, Jaylyn Kelly, Anna Makela, Morgan Sotzen, Cheryl Sisk, Graham Atkin, Rebecca Pratt, Elahé Crockett, Gina Leinninger

**Affiliations:** 1 Department of Physiology, Michigan State University, East Lansing, MI 48824, USA; harr1270@msu.edu (T.H.); ralu.bugescu@gmail.com (R.B.); kellyjay@msu.edu (J.K.); makelaa1@msu.edu (A.M.); sotzenmo@msu.edu (M.S.); 2 Neuroscience Program, Department of Psychology, Michigan State University, East Lansing, MI 48824, USA; sisk@msu.edu; 3 Department of Radiology, Michigan State University, East Lansing, MI 48824, USA; atking@msu.edu; 4 Department of Foundational Medical Studies, Oakland University William Beaumont School of Medicine, Rochester, MI 48309, USA; rebeccapratt@oakland.edu; 5 Department of Medicine, Michigan State University, East Lansing, MI 48824, USA; ecrocket@msu.edu

**Keywords:** lateral hypothalamic area, orexin/hypocretin, delta-like-1 homolog (DLK1), feeding, body weight, anxiety, depression, locomotor activity

## Abstract

Lateral hypothalamic area (LHA) neurons expressing the neuropeptide orexin (OX) are implicated in obesity and anxio-depression. However, these neurons release OX as well as a host of other proteins that might contribute to normal physiology and disease states. We hypothesized that delta-like homolog 1 (DLK1), a protein reported to be co-expressed by all OX neurons, contributes to the regulation of energy balance and/or anxio-depression. Consistent with previous reports, we found that all rat OX neurons co-express DLK1. Yet, in mice and humans only a subset of OX neurons co-expressed DLK1. Since human OX-DLK1 distribution is more similar to mice than rats, mice are a comparable model to assess the human physiologic role of DLK1. We therefore used a viral lesion strategy to selectively delete DLK1 within the LHA of adult mice (DLK1^Null^) to reveal its role in body weight and behavior. Adult-onset DLK1 deletion had no impact on body weight or ingestive behavior. However, DLK1^Null^ mice engaged in more locomotor activity than control mice and had decreased anxiety and depression measured via the elevated plus maze and forced swim tests. These data suggest that DLK1 expression via DLK1-expressing OX neurons primarily contributes to anxio-depression behaviors without impacting body weight.

## 1. Introduction

Obesity is highly coincident with depression and anxiety, suggesting a common biological link between these physiologic and psychiatric diseases. Obese adults have double the risk of developing depression [1,2,3] and depression is associated with increased risk of developing obesity-linked type 2 diabetes [4]. Depression also impedes obesity treatment, as individuals with depression are more likely to engage in excessive consumption of palatable and energy-dense foods but less in physical activity behaviors that prevent weight loss [5,6]. Obesity is linked with anxiety disorders as well [3,7], and diet-induced obesity promotes anxiety-like behavior and stress-induced hypothalamic-pituitary-adrenal (HPA)-axis activation [5]. Treating co-morbid obesity and psychiatric disorders is challenging, however, because antidepressants are less effective in alleviating symptoms for obese individuals [8,9,10]. Rodent obesity models replicate the link of obesity and mood disorders, making them useful to study the cause and connection of the diseases. For example, diet-induced obesity increases depression-like behavior, including reduced exploratory behavior in elevated plus maze (EPM) and open field tests along with increased immobility in the forced swim test (FST) [11]. A high saturated fat diet, similar to that of Western cultures, invokes obesity and also enhances anxiety and depression behaviors in rodents [12]. Thus, while rodents are clinically-relevant models to study coincident obesity and anxio-depression disorders, the molecular underpinnings of these diseases have yet to be defined.

The neuropeptide orexin (OX), also known as hypocretin, may be a common link to obesity and anxio-depression. OX is produced by a small population of neurons found exclusively within the lateral hypothalamic area (LHA) but which project widely throughout the brain [13]. As a result this modest “OX neuron” population exerts an outsized impact on physiology and behavior. The OX peptide is a critical factor for arousal [13], and hence for other wake-dependent behaviors that impact body weight, including feeding and locomotor activity. Due to this linkage, genetic loss of OX peptide causes narcolepsy, but also leads to diminished physical activity and energy expenditure that prompts weight gain and increased risk for type 2 diabetes [14,15,16,17,18,19,20,21,22]. OX may also impact energy balance via modulating sympathetic outflow and brown adipose tissue (BAT) thermogenesis that can beneficially promote oxidative metabolism and weight loss [23,24]. Together, these data support a role for OX neurons in the development of obesity. OX signaling has been separately studied in the context of psychiatric disease, where it promotes anxiety-like behaviors in rodents, such as decreased time spent in the light portion of light-dark box or open arms of the EPM [25,26,27]. By contrast, inhibition of OX signaling or chemogenetic inhibition of OX neurons reduces anxiety and panic-like behaviors [28,29,30,31,32], suggesting contributions of OX and other signals co-expressed by OX neurons. While there is conflicting data on how OX neurons contribute to depression in humans [33,34,35], it is widely accepted that hyperactive OX neuronal signaling contributes to panic disorder [29,31,36]. Thus, strategies to diminish signaling via OX neurons may be useful to treat anxio-depressive disorders, and perhaps obesity. Blunting OX function might seem counterintuitive for promoting weight loss based on reports that genetic deletion of OX reduces arousal, BAT differentiation and thermogenesis that lends to weight gain [37,38,39,40,41,42,43,44]. However, such developmental deletion may derange normal formation of the nervous system and may not reflect the roles of OX neurons in the adult brain. Moreover, loss of OX peptide from intact OX neurons does not alter body weight [38], suggesting that the composite signaling from OX neurons (e.g., other signals released from OX neurons) regulates energy balance. Given the vital role of OX for arousal, however, general blockade of OX signaling may not be ideal for treating obesity and linked psychiatric disorders. Alternatively, blocking specific OX pathways and/or other signals originating from OX neurons might improve anxio-depression while supporting sufficient OX-tone to achieve weight loss without adverse side effects. 

In addition to OX peptide, OX neurons co-express glutamate, dynorphin and proteins such as IGFBP3, neuronal pentraxin (NARP/Nptx2), Lhx9 and the atypical Notch ligand Delta-like homolog 1 (DLK1) [45,46,47,48,49]. Roles of glutamate and dynorphin peptide signaling via OX neurons have been explored [50,51,52] but the roles of the other co-released proteins remain enigmatic. Here we examined the role of DLK1 (also known as pre-adipocyte factor 1 or Pref-1), which is commonly cited as being co-expressed within OX neurons although its function via OX neurons is unknown. DLK1 is an imprinted gene, meaning that only the paternally inherited copy is expressed [53]. While DLK1 is widely expressed in embryonic development and is critical for it [54], there is a much more restricted pattern of DLK1 expression in adults [55]. The function of DLK1 is best understood in adipose development, where it is important for maintaining white adipocytes in an undifferentiated state, and also inhibits thermogenic action in brown adipose tissue [54,56,57,58]. Yet, a role is also emerging for DLK1 in central regulation of energy balance and mood. First, upregulation of genes within the DLK1-DIO3 locus on chromosome 14 are implicated in Prader-Willi syndrome, a developmental disorder in which severe hyperphagia causes children to become obese [59]. Developmental deletion or constitutive over-expression of DLK1 alters anxiety behavior and energy balance in adults [57,60,61] and DLK1 has been implicated in suicide behavior [62]. While most studies have focused on developmental roles of Notch ligands including DLK1, recent work demonstrates a role for Notch signaling in the adult olfactory system and amygdala [63,64]. To our knowledge, these were the first demonstrations that Notch ligands regulate physiology and behavior via the established nervous system. Given that DLK1 remains abundantly expressed in OX neurons of adult rodents [65,66], we hypothesized that DLK1 may contribute to the physiology and behavior regulated by these neurons. Here, we investigated the expression of DLK1 in the LHA during adulthood, and whether it contributes to energy balance and anxio-depressive behaviors attributed to OX neurons.

## 2. Materials and Methods

### 2.1. Animals

**Figure 1:**
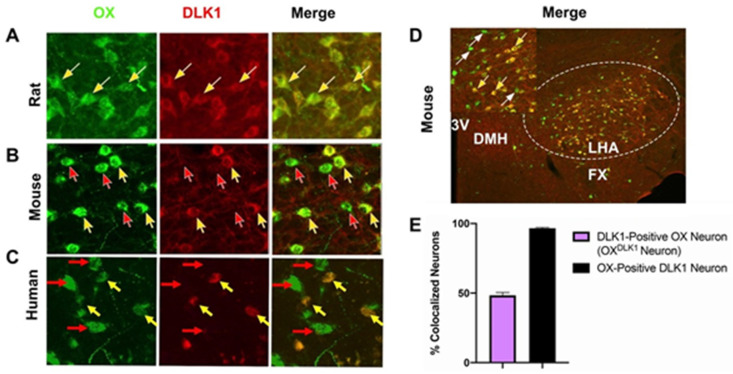
OX (orexin) and DLK1 (delta-like homolog 1) distribution in the LHA (lateral hypothalamic area) differs between species. (**A**) Immunofluorescence for OX (red) and DLK1 (green) in the LHA of rats (*n* = 3), (**B**) mice (*n* = 8), and (**C**) humans (*n* = 3). Yellow arrows identify representative neurons with co-localized OX and DLK1. Red arrows identify neurons with OX immunoreactivity, but not DLK1. OX and DLK1 completely co-localize within the rat LHA, whereas DLK1 is found in approximately half of all OX neurons in the LHA of mice and humans. (**D**) Distribution of OX and DLK1 neurons within the mouse LHA. Neurons with co-expressed OX and DLK1 (yellow arrows) are distributed throughout the region alongside neurons that only contain OX (white arrows). 3V = 3rd ventricle; DMH = dorsomedial hypothalamus; FX = fornix. (**E**) Percentage of OX and DLK1-co-localized neurons in the LHA of mice. Approximately half of all OX neurons are also DLK1 positive (OX^DLK1^) but essentially all DLK1 neurons are OX-positive (*n* = 8).

**Figure 2:**
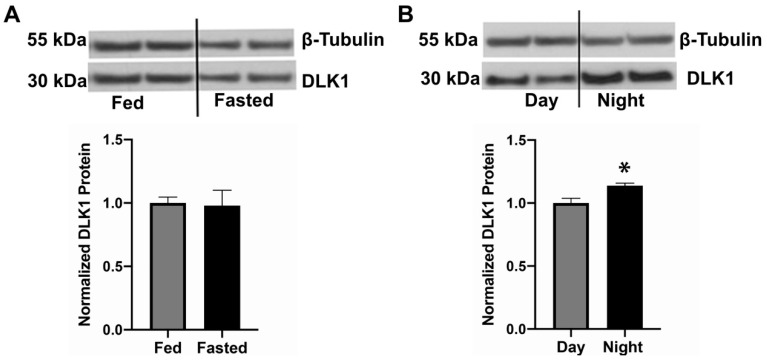
Mouse DLK1 protein expression is regulated in the LHA over the light cycle, but not by energy status. Top: Representative Western Blots of the soluble form of DLK1 (30 kDa) and β-Tubulin (55 kDa, loading control) in mouse LHA. Bottom: Densitometry analysis, which indicates the amount of DLK1 protein in each sample as normalized to ß-Tubulin. (**A**) DLK1 protein expression in the LHA is similar in fed and overnight-fasted mice. (**B**) DLK1 expression in the LHA is increased during the night (ZT15) compared to the day (ZT8). * *p* < 0.05 by Student’s *t*-test, *n* = 3–4 per condition.

Male C57BL/6J mice (The Jackson Laboratory, Stock #000664) and Sprague Dawley rats (Charles River, Strain #400) were used for assessment of Orexin and DLK1 expression (Figure 1 and Figure 2). For all other experiments, we generated study mice by breeding male heterozygous *Dlk1^flox/+^* mice (The Jackson Laboratory, Stock #019074) with female C57BL/6J mice to generate *Dlk1^+/+^* and *Dlk1^flox/+^* mice. Dlk1 is imprinted and is only expressed from the paternal allele, hence we only bred male *Dlk1^flox/+^* mice to generate flox mice, but studied all male and female *Dlk1^flox/+^* progeny similar to previous reports with this line [65]. Our studies were not powered to detect sex differences so data from both sexes was pooled. While we did not observe sex-dependent effects we cannot rule out that they exist, and may account for some of the variability in our behavioral data. Mice were bred and maintained on a 12-h light/12-h dark cycle. Unless specifically noted, mice had ad libitum access to chow (Harlan Tekland #7913), water, nestlets and enrichment habitats. All protocols were approved by the Institutional Animal Care and Use Committee (IACUC) at Michigan State University in accordance with the Association for Assessment and Accreditation of Laboratory Animal Care and National Institutes of Health guidelines. 

Tail biopsies were collected between 2–3 weeks of age to genotype all potential study animals. Extracted tail DNA was analyzed via polymerase chain reaction (PCR) with the following primers to identify *Dlk1^flox/+^* mice for studies. 15412: 5′ AGA TTC CCC CAC CTC CAA C 3′; 15413: 5′ TTC CCA AAC TGG ACA TGA GC 3′.

### 2.2. Human LHA Tissue

Brain tissue dissections were supported by generous donations to the Michigan State University Willed Body Program. Tissues were held in fixative for up to 16 years. “The Human Brain Atlas” (http://brains.anatomy.msu.edu/brains/human/index.html) was used to guide dissection of tissue from the LHA from three neurologically normal adult brains. A freezing microtome was used to generate four series of 30 µm sections from each sample, one of which was analyzed for DLK1 and OX via immunofluorescence, as described below.

### 2.3. Immunofluorescence

Rats were anesthetized and perfused with 4% paraformaldehyde (PFA). At Zeitgeber-Time 15 (ZT15, 3 h post lights off) mice were anesthetized via intraperitoneal (i.p.) pentobarbital overdose, then were perfused with 4% PFA ± 0.4% picric acid consistent with prior methods [65,66]. Both fixatives yielded comparable percentages of DLK1-positive neurons (data not shown), but addition of picric acid improved the signal-to-noise ratio and analysis, so this fixation method was used for all mouse images shown herein. Brains were removed and post-fixed overnight in the same fixative, followed by dehydration in 30% sucrose/PBS. Brains were sectioned coronally at 30 µm into four equal series spanning the entire brain, and one series was utilized for immunofluorescence. For detection of DLK1 in mouse and human LHA, we first performed antigen retrieval as described by Meister et al. [65]. Briefly, sections underwent three 8-min PBS washes followed by 30 min incubation in 10 mM sodium citrate solution with 0.05% Tween 20 at 80 °C. After three PBS rinses, all brain sections were blocked in normal donkey serum (Jackson ImmunoResearch, #017-000-121) and exposed to primary antibodies per our published protocol [67]. Primary antibodies included DLK1 (mouse, 1:100, sc-376755) and Orexin (goat, 1:1000, sc-8070) from Santa Cruz Biotechnology, Dallas, TX, USA). For detection, sections were exposed to species-specific secondary antibodies conjugated to Alexa-568 (1:200, cat# AB_2534017, Thermo Fisher, Waltham, MA, USA) or Alexa-488 (1:200, #703-545-155, Jackson ImmunoResearch, West Grove, PA, USA). Sections were mounted onto slides, coverslipped and analyzed using an Olympus BX53 fluorescence microscope outfitted with FITC and Texas Red filters. Images were collected using Cell Sens software and a Qi-Click 12 Bit cooled camera, and analyzed using Photoshop software (Adobe). Any adjustments of brightness and/or contrast were applied uniformly to all samples. Counts of Orexin and DLK1 were performed from all Orexin-containing sections. Briefly, 10× microscopy images were viewed in Photoshop and used to count each co-labeled neuron, as well as neurons labeled for only DLK1 or only OX. Graphed data represents the average percentage of co-localized DLK1 and OX neurons, *n* = 8 mice. 

### 2.4. Western Blotting

Adult age-matched C57/BL/6J male mice were fed ad libitum or fasted for 24 h (*n* = 3 per condition). Concurrently, adult age-matched C57/BL/6J male mice were euthanized either during the day (ZT8) (*n* = 3) or at night (ZT15) (*n* = 4). The brains were placed in a stainless-steel brain matrix on ice and used to microdissect out the LHA, which was snap-frozen on dry ice and stored at 80 °C. Samples were lysed in RIPA Lysis Buffer and protein concentrations were determined using a BCA protein assay kit. Equivalent volume samples were separated on BOLT 10% Bis-Tris Plus gels and transferred onto PVDF membrane. The membranes were rinsed with TBST, incubated in blocking solution and then incubated overnight at 4 °C in blocking solution and antibodies against DLK1 (mouse, 1:100, sc-376755Santa Cruz Biotechnology, Dallas, TX, USA).) and Beta-Tubulin (rabbit, 04-1049, MilliporeSigma, Burlington, MA, USA). The predominant form of DLK1 in the LHA is a cleaved, soluble protein presenting at 30 kDa, consistent with prior reports of DLK1 in brain tissue [68]. Beta-Tubulin (55 kDa) was used as a loading control to check that all lanes in the gel contain the same amount of sample and to verify the amount of protein present in the sample. Membranes were incubated in HRP-conjugated secondary antibodies diluted in 5% non-fat milk/1× TBST for 1 h at room temperature, followed by washes and chemiluminescent detection (Advansta, Thermo Fisher, Waltham, MA, USA). Image J software was used to analyze densitometry. 

### 2.5. Preparation of Control and DLK1^Null^ Mice for Studies

Male and female *Dlk1^flox/+^* mice (8–10 weeks) received meloxicam (5 mg/kg, s.c.) prior to stereotaxic injection of either AAV1-hSyn-H1-eGFP-Cre (subsequently referred to as AAVcreGFP, University of Pennsylvania Vector Core) or AAVeGFP virus into each side of the LHA (400 nL per side, A/P: −1.34, M/L: ±1.05, D/V: −5.2). For gene expression analysis, mice received either AAVeGFP (Control *n* = 5) or AAVcreGFP (DLK1^Null^ *n* = 6); then, 8 weeks later, the LHA was collected from each side of the brain as previously described [69]. Each LHA hemisphere was analyzed as a separate sample. RNA was extracted using TRIzol (Thermo Fisher, Waltham, MA, USA) and 200 ng from each sample was converted to cDNA using the SuperScript first-strand synthesis system for RT-PCR (Invitrogen). Sample cDNAs were analyzed in triplicate via quantitative RT-PCR for gene expression of *Dlk1*, *Ox* and *Gapdh* (internal control) using TaqMan reagents (Invitrogen). Fold expression was calculated relative to AAVeGFP injected LHA samples (control) using the 2^−∆∆Ct^ method. Three samples did not yield sufficient RNA for analysis and were excluded, resulting in Control *n* = 8, DLK1^Null^ *n* = 11.

Separate cohorts of mice received LHA injections, then were individually housed and assessed weekly for food intake and body weight over 8 weeks. At 12 weeks post-surgery, the mice were analyzed via metabolic phenotyping, followed by a battery of behavioral tests (sucrose preference, open field, elevated plus maze, forced swim test) as equipment and scheduling permitted. As a result, mice underwent behavioral testing at different ages, but we did not observe any behavioral differences suggestive of age-dependent effects. All studies were completed by 42 weeks post-surgery (52 weeks of age), at which point, the mice were perfused with fixative and brains were assessed via immunofluorescence for DLK1, as described above. DLK1^Null^ mice were only included in the final study data if post hoc immunofluorescence microscopy confirmed loss of DLK1 confined to the LHA. Controls were only included if DLK1 was intact in the LHA. This resulted in Control *n* = 7, DLK1^Null^ *n* = 15 mice for all metabolic and behavioral datasets.

### 2.6. Metabolic Phenotyping

Body composition was measured 12 weeks post-surgery using a nuclear magnetic resonance-based instrument (Minispec mq7.5, Bruker Optics, Billerica, MA, USA). Mice were then acclimated in TSE Cages (PhenoMaster, TSE Systems, Chesterfield, MT, USA) for 24 h, followed by 3 days of continuous measurement of food and water intake, locomotor activity, energy expenditure and respiratory exchange ratio (RER). The final 24-h cycle was used for data analysis. Ambient temperature was maintained at 20–23 °C and the airflow rate through the chambers was adjusted to maintain an oxygen differential around 0.3% at resting conditions.

### 2.7. Sucrose Preference

Mice were single-housed for at least 1 week prior to 2-bottle choice testing for water vs. 1% sucrose solution as described previously [67]. Graphed data represent the average amount of consumed sucrose, water consumed, and sucrose preference over 2 days. 

### 2.8. Open Field and Amphetamine-Induced Locomotor Activity

Locomotor activity was assessed during the light cycle in open field chambers, with an overhead digital CCD camera and video tracking software (CleverSys). First, to test novelty-induced locomotor activity, each mouse received an i.p. injection of PBS, was placed into the middle of the chamber and activity was tracked for 30 min. Mice were then treated with D-amphetamine hydrochloride (4 mg/kg, i.p., Cayman Chemical, #14204), placed back in the chambers and monitored for an additional 60 min. 

### 2.9. Elevated Plus Maze (EPM)

Mice were acclimated for 1 h in the testing room under red light. For testing, a mouse was placed in the middle of the maze with its head pointed toward a closed arm. Ambulatory movement was tracked for 5 min using an overhead CCD camera and video tracking software (CleverSys, Reston, VA, USA). The maze was sanitized between each test. Graphed data represent the average percentage of their time spent in the open or closed arms of the maze. 

### 2.10. Forced Swim Test

Each mouse was placed in a cylinder of room temperature water and filmed via an overhead CCD camera. Mice were removed after 240 s and returned to their home cage. Videos were viewed and manually scored to measure the amount of time spent mobile, which was defined as any movements other than those necessary to balance the body and keep the head above the water. While it is possible to measure the immobility time directly, we found it easier to detect and measure active movements rather than the lack of such movements. Immobility time was then calculated by subtracting the total amount of mobility time from the total test time. 

### 2.11. Data Analysis

Unpaired Student’s *t*-tests, one-way analysis of variance (ANOVA), and area under the curve (AUC) analyses were calculated using GraphPad Prism (GraphPad Software Inc., San Diego, CA, USA). Error bars depict ± SEM. The alpha value for statistical analysis was 0.05 and differences were considered to be significant for *p* < 0.05.

## 3. Results 

### 3.1. DLK1 Protein Is Expressed in a Subset of Orexin Neurons in Mice

First, immunofluorescence microscopy was used to assess the distribution of DLK1 protein within OX neurons of the LHA. Previous reports from rats indicated that DLK1 is present in all OX neurons [65], and we confirmed complete overlap of DLK1 and OX protein expression in Sprague Dawley rats (Figure 1A, yellow arrows identify representative DLK1 + OX neurons). In mice, however, this method only identified DLK1 within a subset of OX neurons (Figure 1B, yellow arrows). Mice also had OX neurons that did not contain bright DLK1 immunoreactivity (Figure 1B, red arrows). These species differences in the distribution/abundance of DLK1 in OX neurons prompted us to analyze human post-mortem LHA tissue to determine if the human distribution of DLK1 protein within OX neurons is similar to mice or rats. Immunofluorescent analysis of human LHA demonstrated that DLK1 is present in some, but not all, OX-expressing neurons (Figure 1C, yellow arrows). As in mice, human LHA tissue also contained some OX-labeled neurons that did contain DLK1 (Figure 1C, red arrows). Taken together, these data suggest that mice are a closer model of the DLK1 distribution found in human OX neurons, and thus are more clinically relevant for probing the role of DLK1 in LHA OX neurons. We therefore assessed the extent of DLK1 and OX co-immunoreactivity within the mouse LHA. DLK1 was equally distributed throughout the mouse LHA and was not associated with any particular subregion of OX-expressing neurons (Figure 1D). Nearly all DLK1-expressing neurons also contain OX, whereas approximately half of all OX-expressing neurons contained visible, high levels of DLK1 protein (Figure 1E). We subsequently refer to mouse OX neurons that co-express DLK1 as “OX^DLK1^ neurons” to differentiate them from the neurons that only contain OX and not DLK1 (“OX^Only^ neurons”). 

### 3.2. DLK1 Protein Expression Is Regulated in the LHA

OX neurons have been implicated in energy balance and arousal, and OX expression is increased by fasting and during times of enhanced arousal/alertness [70]. We reasoned that if DLK1 expressed within OX^DLK1^ neurons contributed to these facets of physiology, then DLK1 expression might also be regulated by energy status or during time periods typical of sleeping vs. wakefulness. DLK1 exists in a cleaved and soluble 30 kDa form in the brain [65], which we similarly observed within the LHA (Figure 2A,B). Fed and fasted mice exhibited similar DLK1 protein expression within the LHA, suggesting that energy status does not alter DLK1 expression (Figure 2A). However, DLK1 protein expression was upregulated in the LHA during the night compared to the day (Figure 2B, Day = 1.00 ± 0.04, Night = 1.14 ± 0.02, *p* < 0.05). Hence, the regulation of LHA DLK1 protein expression across the light/dark cycle in the LHA suggests that DLK1 may play a role in arousal/alertness behaviors that are known to be modulated via OX neurons. 

### 3.3. Genetic Deletion of DLK1 from the LHA of Adult Mice Does Not Compromise OX Expression

**Figure 3:**
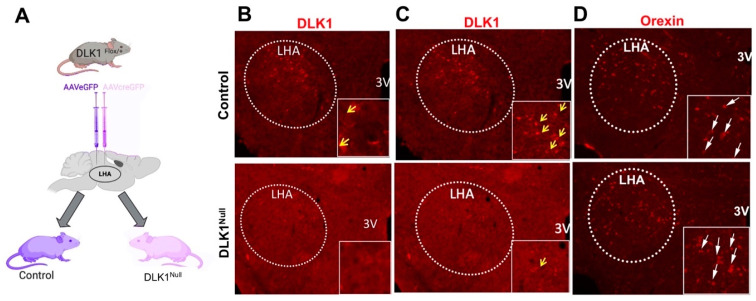
LHA-specific deletion of DLK1 in adult mice. (**A**) DLK1^Flox/+^ mice received bilateral LHA injections of either AAVeGFP (yielding Control mice with intact DLK1) or AAVcreGFP (to yield mice with cre-lox mediated deletion of DLK1 from the LHA, DLK1^Null^ mice). (**B**) Immunofluorescence for DLK1 (red) in the LHA confirms intact DLK1 in Controls compared to significant reductions in DLK1 in DLK1^Null^ mice by 2 weeks after AAV injection (*n* = 3). (**C**) Analysis at the end of the metabolic and behavioral phenotyping (30–42 weeks post-surgery) confirms that Control mice retain DLK1 expression, but DLK1^Null^ mice lack DLK1 expression in the LHA. (**D**) OX immunoreactivity remains intact within the LHA of Control and DLK1^Null^ mice at the end of the study. For (**C**,**D**), Control *n* = 7, DLK1^Null^ = 15.

**Figure 4:**
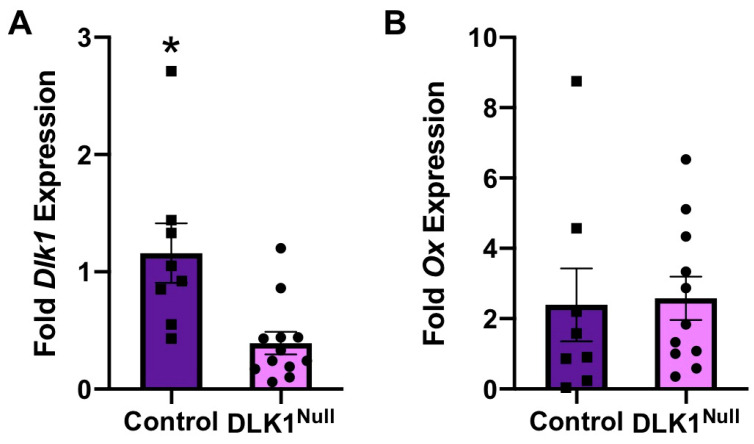
*Dlk1*, but not *Ox*, is depleted in the LHA of adult DLK1^Null^ mice. (**A**) Control mice have significantly higher fold mRNA expression of *Dlk1* within the LHA compared to DLK1^Null^ mice. (**B**) Control and DLK1^Null^ mice have comparable *Ox* gene expression within the LHA. Black squares identify individual Control samples. Black dots represent the individual DLK1^Null^ samples.* *p* < 0.05. Control *n* = 8, DLK1^Null^ = 11.

Given that DLK1 protein is strongly expressed within a subset of OX neurons, and is regulated by physiological stimuli, we hypothesized that DLK1 contributes to the physiology mediated via OX neurons. To explore the necessity for DLK1 in the adult mouse brain, we used a viral-genetic method to site-specifically deplete DLK1 in the LHA of adult mice (Figure 3A). Adult DLK1^flox/+^ mice received AAVeGFP in the LHA so as to leave DLK1 intact (Control mice). DLK1^flox/+^ littermates were injected with AAVcreGFP, whereby cre-mediated recombination deleted DLK1 expression within the LHA (referred to as DLK1^Null^ mice). As DLK1 is paternally expressed, we only used DLK1^flox/+^ mice that inherited the DLK1 gene from the sire, but were able to study all heterozygous progeny consistent with prior studies using this line [71]. Within 2 weeks of AAV injection, this method depleted DLK1 within the LHA of DLK1^Null^ mice compared to controls (Figure 3B). We therefore generated cohorts of Control and DLK1^Null^ mice, assessed them via a battery of metabolic and behavioral phenotyping and then examined their brains for DLK1 expression. Post hoc analysis confirmed that these Control mice retained LHA DLK1 protein expression throughout the duration of our studies, but DLK1^Null^ mice had visibly reduced DLK1 expression (Figure 3C). However, Control and DLK1^Null^ mice exhibited similar expression of OX throughout the study, indicating that the genetic depletion method did not reduce DLK1 secondary to damage/ablation of OX neurons (Figure 3D). In a separate cohort of mice analyzed for LHA gene expression, the DLK1^Null^ mice had significantly less *Dlk1* expression compared to Controls (Figure 4A) but retained normal *Ox* expression (Figure 4B). Taken together, these data confirm that our viral deletion strategy specifically depleted DLK1 within the LHA of adult mice without compromising OX expression or the viability of the neurons. Thus, comparing Control and DLK1^Null^ mice can reveal the contribution of LHA DLK1 to physiology and behavior. 

### 3.4. Genetic Deletion of DLK1 from the LHA Does Not Alter Energy Balance

**Figure 5:**
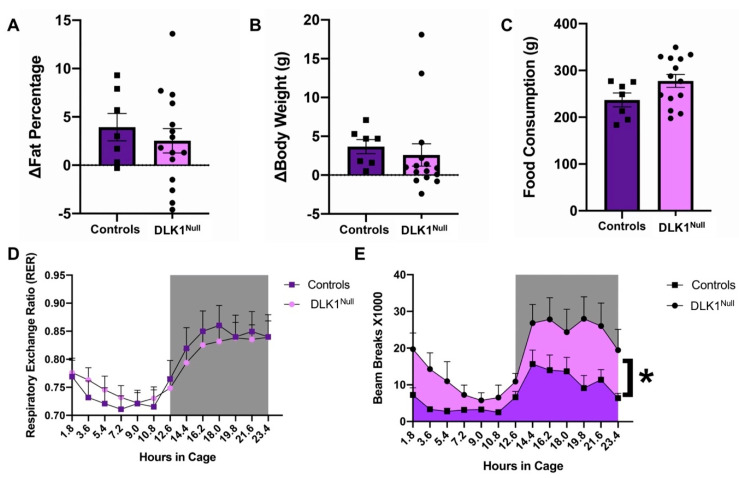
DLK1-deletion in the LHA increases locomotor activity but does not alter energy balance. Control and DLK1^Null^ mice exhibited similar (**A**) change (∆) in total body fat percentage, (**B**) body weight and (**C**) the grams of food consumed over 8 weeks. (**D**) Control and DLK1^Null^ mice had similar RER over the light/dark cycle. (**E**) Area under the curve analysis indicates that DLK1^Null^ mice exhibit more ambulatory locomotor activity in metabolic cages than Control mice, * *p* < 0.05. For (**D**,**E**), gray shading identifies the dark cycle. Control *n* = 7, DLK1^Null^ = 15.

Given the important contribution of OX neurons to maintaining energy balance, and that DLK1 is expressed in approximately half of all OX neurons, we reasoned that loss of LHA DLK1 might impact energy homeostasis. However, we found no differences in the percentage of body fat, body weight or food consumption between Control and DLK1^Null^ mice (Figure 5A–C). Control and DLK1^Null^ mice had similar metabolic profiles in terms of RER (Figure 5D). Thus, despite the vital role of OX neurons for energy balance, and that DLK1 is expressed in the OX^DLK1^ subset, DLK1 expression is not necessary for regulation of energy homeostasis. Interestingly, DLK1^Null^ mice engaged in significantly more locomotor activity than Control mice while in metabolic cages (Figure 5E, *p* < 0.05), but this did not lead to elevated energy expenditure. This finding suggests that DLK1 may contribute to behaviors that require locomotor activity, although the movement is not sufficient to modify metabolism and body weight.

### 3.5. Genetic Deletion of DLK1 from the LHA Does Not Alter Reward Behavior

**Figure 6:**
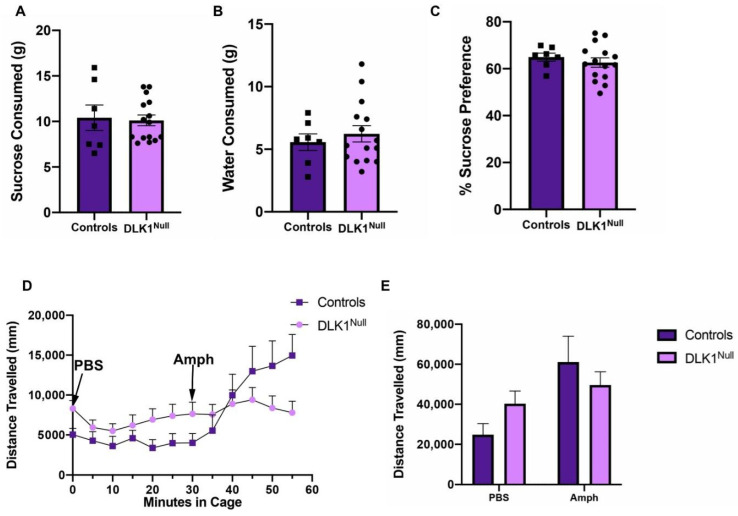
DLK1-deletion in the LHA does not alter natural reward behaviors. The amount of (**A**) 1% sucrose solution and (**B**) water consumed over 2 days in a two-bottle test. Bottles were weighed to assess consumption so values are expressed in grams (g). (**C**) DLK1^Null^ mice and control animals exhibit similar sucrose preference. (**D**) Distance traveled (mm) by mice in open field boxes over 60 min. Data points represent 5 min bins. Mice received a PBS injection at *t* = 0 min and an amphetamine injection at *t* = 30 min. **(E)** Total distance traveled (mm) after PBS and amphetamine injection. For (**A**–**C**), Control *n* = 7, DLK1^Null^ = 15. For (**D**,**E**), Control *n* = 6, DLK1^Null^ = 14.

OX neurons modulate goal-directed behaviors to obtain natural and drug rewards [70,72], hence we reasoned that loss of DLK1 expression from OX^DLK1^ neurons might impact reward behaviors. However, loss of LHA DLK1 did not alter the amount of palatable sucrose solution (a natural reward) or water consumed, nor did it alter sucrose preference in DLK1^Null^ mice compared to Controls (Figure 6A–C). Since OX neurons are well established to project to and modulate the activity of dopamine neurons that govern reward behaviors [72,73], we next examined whether loss of LHA DLK1 (including from OX^DLK1^ neurons) impacted the mesolimbic dopamine system. To test this, Control and DLK1^Null^ mice were treated with amphetamine, which causes release of all mesolimbic dopamine into the synaptic cleft and produces locomotor activity commensurate with the function of the dopamine system. Amphetamine-induced locomotor activity assessed in open field chambers was not significantly different between Control and DLK1^Null^ mice (Figure 6D,E). These data suggest that DLK1 expression in the LHA is not required for natural reward behavior or dopamine-mediated locomotor activity.

### 3.6. Genetic Deletion of DLK1 from LHA Neurons Diminishes Anxiety Behavior

**Figure 7:**
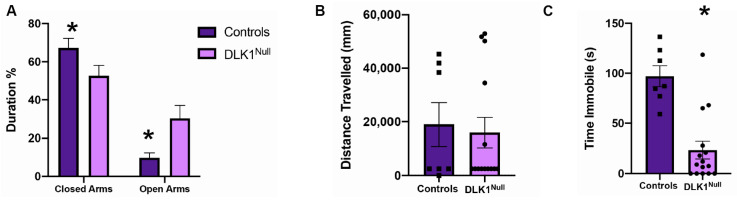
DLK1-deletion in the LHA decreases anxiety and depression-like behavior in mice. (**A**) Percentage of time spent in open and closed arms of the elevated plus maze. * *p* < 0.05 by one-way ANOVA. (**B**) Average distance traveled (mm) in the open field boxes over 24 min (3–27 min). This period was chosen to exclude the initial and final minutes, during which the experimenter was leaving/entering the testing room, as this activity could have disturbed the mice and their activity. (**C**) Time (s) spent immobile during the forced swim test, * *p* < 0.05 by Student’s *t*-test. Control *n* = 7, DLK1^Null^ = 15.

OX neurons are implicated in anxiety and depression, hence we explored whether loss of DLK1 expression from OX^DLK1^ neurons might contribute to these states. As expected, Control mice spent more time in the closed arms of the EPM vs. the open arms, as the open areas are considered to be an anxiety-producing environment. In contrast, DLK1^Null^ mice spent more time in the open arms of the EPM compared to Controls, suggesting that loss of LHA DLK1 might reduce anxiety behavior (Figure 7A, Control Closed Arm = 9.8 ± 2.5% vs. DLK1^Null^ Closed Arm= 52.6 ± 5.5%; Control Open Arm = 67.2 ± 5.1% vs. DLK1^Null^ Open Arm = 30.3 ± 6.8%, *p* < 0.05). However, given the increased locomotor activity of DLK1^Null^ mice in home cage-like metabolic cages, we reasoned that DLK1^Null^ mice might have spent more time in the open arms simply because they move more. To explore this possibility, we assessed locomotor activity in open field boxes, which have been used to characterize hyper-locomotor phenotypes. We did not observe any significant differences in the time Control and DLK1^Null^ mice spent in the center vs. the periphery of the boxes (not shown) nor in their overall distance traveled (Figure 7B). These data suggest that lacking LHA DLK1 does not promote excess locomotor activity in a novel environment, and hence, the increased open arm activity in the EPM is not a side effect of general hyperactivity. Lastly, we tested Control and DLK1^Null^ mice via the forced swim test, in which time spent immobile is indicative of anxious or depressed behavior. DLK1^Null^ mice spent significantly less time immobile than Control mice (Figure 6C, Control = 97.1 ± 10.41, DLK1^Null^ = 23.6 ± 8.9, *p* < 0.0001). In sum, these data suggest that LHA DLK1 plays an anxio-depressive role, and that decreasing DLK1 expression in the LHA can reduce anxiety and depression behavior.

## 4. Discussion

OX neurons are specific to the LHA and are defined by their expression of OX peptide, which contributes to energy balance and anxio-depression. Yet, OX neurons also contain myriad releasable proteins that might contribute to these processes. Here, we explored how one such protein, DLK1, is co-expressed with OX in the adult brain, and then performed LHA-specific depletion to assess its necessity for physiology and behavior. Our data suggest that DLK1 in the established LHA contributes to anxio-depression but not energy balance. These data are, to our knowledge, the first to describe a functional role for DLK1 in the LHA, and suggest that modulating DLK1 may mediate specific aspects of the diverse physiology attributed to OX neurons. 

Our findings challenge the prevailing view that all OX neurons co-express DLK1 and reveal species differences in OX-DLK1 distribution. Prior reports demonstrated that DLK1 is expressed in the LHA of rats [65], where it is co-expressed in all OX neurons but not in other LHA neurons [65]. Using the same immunofluorescence technique, we also found that all rat OX neurons contained DLK1, and did not observe any LHA DLK1 neurons that lacked OX immunoreactivity. We therefore intended to use DLK1 as a proxy to detect all “OX neurons” in another study [70], so that we could detect neurons even after deleting OX expression. To our surprise, using the same DLK1 detection method in mice only revealed DLK1 expression in half of the OX neuronal population. We consistently observed this distribution of DLK1 in ~50% of OX neurons with various methods of fixation, with or without antigen retrieval treatment, and using multiple different antibodies against DLK1 (data not shown). Moreover, an independent report concurs that in mice the DLK1 protein is expressed in some, but not all, OX neurons [49]. The only methodological difference between our study and prior DLK1 immunolabeling studies is that we did not treat any of the rodents with colchicine prior to fixation. Colchicine blocks axonal transport to enhance the concentration of releasable proteins within soma so that they can be detected, and we concur that colchicine treatment is absolutely required to detect some LHA peptides that are not typically abundant within soma (for example, neurotensin) [70,74]. However, since prior authors reported that colchicine had no effect on the cellular distribution of DLK1 [65] and was not needed to concentrate DLK1 in the soma, there was no physiological rationale to use it. Since colchicine can also invoke cellular stress that artificially upregulates peptide expression, we did not want to potentially introduce this experimental artifact. Moreover, our data confirm that colchicine is not necessary for detection of DLK1 because we found identical, complete co-localization of DLK1 and OX in the brains of untreated rats, just as was reported in colchicine-treated rats. These findings support that rats and mice have different physiologic distributions of LHA DLK1 protein expression. This species difference is interesting but is not specific to DLK1: gene expression differs considerably between rats and mice as documented in previously published studies [74,75,76,77]. Given these species differences, we examined whether human LHA reflected the DLK1 distribution of rats, mice or neither. We found that the distribution of DLK1-OX in human LHA is more similar to mice than rats, suggesting that mice are the preferable rodent model to mimic the OX neuronal system within humans and to explore its function.

It has long been hypothesized that there are distinct populations of OX neurons within the LHA, and subsets of OX neurons have recently been distinguished via their electrophysiological signatures or connectivity [78,79]. Despite these functional differences, there was no known biological marker for distinguishing OX populations [80]. The enrichment of DLK1 protein in approximately half of all mouse OX neurons suggests that DLK1 may be used as a marker to distinguish between OX neuron populations in mice. Likewise, DLK1 has been used to distinguish other neuronal subgroups, including a subset of motor neurons with fast-acting biophysical properties [81]. However, DLK1 transcripts have been detected in all mouse OX neurons, and in many non-OX neurons [78]; this would seem to contradict our protein expression results and argues against DLK1 as a molecular marker of OX neuronal subsets. One possible explanation for this discrepancy is that all OX neurons may transcribe DLK1, but a translational or post-translational mechanism may only allow the protein to be expressed in certain OX neurons. Alternatively, we cannot rule out that there are differing levels of DLK1 expression in mouse OX neurons, such that neurons with high expression were readily detectable via immunofluorescence while those with low DLK1 expression could not be detected. Should this be true, such sizable differences in protein expression would still suggest functional differences between OX neurons (e.g., DLK1 high-expressing and low-expressing OX neurons), and hence, vouch that there are OX-DLK1 specified subsets.

DLK1 is commonly cited as a co-expressed protein within LHA OX neurons, but to our knowledge, ours is the first study to examine its functional role in the adult LHA. Here, we combined the use of the cre-lox system and site-specific AAV injection to selectively deplete DLK1 within the LHA of adult mice so as to reveal its physiologic necessity. Given that DLK1 is expressed within OX neurons, we reasoned that it might impact two important branches of physiology attributed to OX neurons: energy balance and behavior. Likewise, global, developmental deletion of DLK1 has been linked with obesity and anxio-depression [57,59,60,61]. Indeed, DLK1 is extremely important in predipocytes, and contributes to peripheral regulation of adipose tissue [54,56,57,58]. However, our findings indicate that LHA DLK1 is not essential for centrally-regulated energy balance in adult mice, as Control and adult DLK1^Null^ mice demonstrated similar feeding, metabolic profiles, body composition (not shown) and body weight. Similarly, DLK1^Null^ mice did not exhibit any alterations in reward behaviors, such as sucrose preference or amphetamine-induced locomotor activity, which might have implicated a disrupted LHA → mesolimbic circuit that could alter feeding and body weight. While OX neurons have been linked to reward behavior [31,72,80], these data suggest that DLK1 expressed by OX neurons is not key for reward signaling. We note that our studies were only performed in chow-fed mice, and in some cases, altered control of energy balance is not apparent until animals are challenged with a palatable, high-fat diet. However, the absence of any disruption of chow feeding, metabolism or body weight, all of which are perturbed by alterations in OX expression, suggest that the DLK1 co-expressed by OX neurons is not a primary modulator of body weight.

We also examined the role of LHA DLK1 within psychiatric behavior, given that DLK1 has been examined previously in the context of anxio-depression using mouse models [56,65,68,71]. However, global, developmental deletion of DLK1 has yielded equivocal phenotypes, either increasing [60,61,82] or having no effect on anxiety behavior. DLK1 is broadly expressed in the brain, so it is possible that early deletion may impede development and function of many neuronal circuits that modulate behavior (in the LHA and elsewhere.) By contrast, we examined the role of DLK1 within the LHA of adult mice, a site that retains robust DLK1 expression. Adult-onset deletion of DLK1 reduced anxiety and depression behavior in mice, supporting a role for LHA DLK1 in behavior. Intriguingly, DLK1 expressed within a subset of mouse central amygdala neurons has been shown to contribute to fear behavior [63,79]. Taken together, these data and our current findings suggest that DLK1 might exert a pro-anxiety/depression/fear role, and that decreasing DLK1 expression could dampen these responses. OX neurons, and OX peptide specifically, have been implicated in anxiety, panic behavior [28,29,30,31,32] and depression [33] via increasing signaling to various areas of the limbic system [82,83]. Given that DLK1 is co-expressed in a subset of mouse OX neurons, our data suggest that DLK1 protein may also contribute to anxio-depressive behaviors. Notably, the phenotypes we observed after adult-onset deletion of LHA DLK1 are not secondary to alterations in OX, as confirmed by the similar OX expression in Control and DLK1^Null^ mice even a year after DLK1-depletion. Hence, OX and DLK1 may both contribute to anxiety behavior. Given that DLK1 is only co-expressed by a subset of OX neurons, it will be important to determine if these neurons exert distinct, DLK1-amplified behavioral responses to anxiety compared to OX neurons that lack DLK1. For example, it is possible that OX^DLK1^ neurons and OX^Only^ neurons differ in DLK1 expression and projection targets, and thus might modulate anxio-depressive behavior via different anatomical and signaling mechanisms. Our findings of a mouse DLK1-defined subset of OX neurons may enable future studies to parse the roles of OX neurons in anxio-depressive behavior.

## 5. Conclusions

OX neurons are implicated in the pathogenesis of adult-onset obesity and anxio-depression, and have deservedly received attention for their roles in these diseases. Yet, the function of other proteins co-expressed within OX neurons have been comparatively ignored. Our data suggest that DLK1 is not just an inert co-expressed protein in all OX neurons, but may in fact contribute to some of the physiology attributed to OX neurons. Our finding that deleting DLK1 from adult OX neurons decreases anxiety behavior without altering energy balance suggests that DLK1 contributes to specific aspects of physiology and behavior. Going forward, understanding the DLK1 system might suggest mechanisms to modulate DLK1-mediated anxio-depression that avoid altering body weight. Indeed, since DLK1 is a useful protein marker to parse OX neurons in mice and humans, it will be of value to examine if the DLK1-defined subset aligns with one of the previously identified projection and/or electrophysiology-defined groups of OX neurons. If true, this finding could pave the way for genetic approaches to selectively modulate specific OX subsets to discern their functions. Further studies to examine the role of DLK1 in the adult LHA will be useful to understand the function of OX neurons, as well as the development of anxio-depressive disorders and to elucidate potential treatments for them.
